# Temporally disjunct herbaceous species differ in leaf embolism resistance

**DOI:** 10.1111/nph.70335

**Published:** 2025-07-01

**Authors:** Ian M. Rimer, Scott A. M. McAdam

**Affiliations:** ^1^ Department of Botany and Plant Pathology Purdue University West Lafayette IN 47907 USA

**Keywords:** embolism, flowering, herbaceous, phenology, phylogeny, stomata, xylem

## Abstract

- In the temperate Northern Hemisphere, herbaceous community composition undergoes major seasonal phenological shifts. Despite significant variation in water availability across growing seasons, few studies have associated physiological traits with seasonal shifts in community composition.
- Key ecophysiological traits, including leaf embolism resistance, were measured in 41 phylogenetically diverse herbaceous species native to the central forest‐grassland ecotone of North America. Traits were tracked monthly in *Solidago canadensis* L. to explore seasonal plasticity. Correlations between ecophysiological traits and flowering time, plant height, and ecological guild were examined. Further analyses were conducted to investigate if traits were constrained by phylogeny or seasonal differences in climatic variables.
- Taller and later flowering species had more embolism‐resistant leaf xylem and greater stomatal safety margins (SSM) than smaller, spring‐flowering species. We found no seasonal variation in traits in *S. canadensis*, suggesting minimal seasonal phenotypic plasticity in these traits. No phylogenetic signal was found for any trait suggesting that climate rather than phylogeny drives variation in ecophysiological traits in herbs.
- We conclude that leaf embolism resistance and SSM are adaptively relevant traits associated with the phenological differentiation of temporally disjunct herbaceous species. The association of leaf embolism resistance and phenology has implications for herbaceous community adaptation to changing climates.

## Introduction

Phenological shifts in the herbaceous flora of the temperate Northern Hemisphere have captivated humans for as long as plants have been described in written form (Theophrastus *et al*., 1978). In many of these temperate ecosystems, major seasonal shifts in herbaceous species community composition are associated with seasonal changes in phenology (Poppenwimer *et al*., 2023). Floristic transitions in the herbaceous community of the Northern Hemisphere provide essential resources for pollinators and herbivores throughout the season, thus ultimately maintaining ecosystem stability and resilience (Roberts, 2004). Flowering time varies dramatically across herbaceous angiosperms, with particular syndromes characterizing these temporally unique floras, albeit with some exceptions (Schemske, 1977; Nagahama & Yahara, 2019). The spring flora consists of some of the first herbaceous species to emerge after winter and is largely ephemeral, with most species rapidly completing flowering and seed‐set before the closing of a forest canopy, after which leaf senescence, death, or dormancy follows (Vézina & Grandtner, 1965; Lapointe, 2001). By late‐summer, the herbaceous flora has transitioned to a suite of species that are scarcely present above ground in the spring (Gaudinier & Blackman, 2020).

This shift in species composition and flowering time over seasons has been well documented (Sparks & Menzel, 2002; Murphy & McCarthy, 2014), having both a phylogenetic signal and being under genetic control by temperature and day length signals (Putterill *et al*., 2004; Bernier & Périlleux, 2005). Phenological shifts in the flora are believed to be maintained by selection for synchrony in sexual reproduction and escaping unfavorable conditions for flowering (Bernier, 1988). A phenological dependence on day length means climatic and seasonal changes in precipitation and temperature regimes can potentially imperil species (Cleland *et al*., 2007; Freimuth *et al*., 2022; Vitasse *et al*., 2022). While reproduction plays a role in determining phenological syndrome, due to the timing of plant and pollinator interactions (Sargent & Ackerly, 2008; Mitchell *et al*., 2009; Freimuth *et al*., 2022), water availability at different times of the year might also provide a strong selective pressure on herbaceous species phenology (Lens *et al*., 2013; Brodribb *et al*., 2020). The association between seasonal water availability, drought resistance, and phenology has yet to be explored.

Water transport in plants is driven by a pressure gradient generated by the evaporation of water at the leaf surface, pulling water from the soil through the xylem under tension (Buckley *et al*., 2017; Venturas *et al*., 2017). Water transport under tension causes the xylem to be susceptible to air infiltration when tension increase, forming an embolism that blocks further water transport (Sperry & Tyree, 1988; Tyree & Sperry, 1989; Lens *et al*., 2013; Brodribb *et al*., 2016). Once a majority of the xylem conduits have embolized (> 50%), hydraulic failure ensues, leading to leaf and plant mortality (Cardoso *et al*., 2022; Brodersen *et al*., 2024). Embolism resistance of the xylem is a critical trait linked to plant drought tolerance (Tyree & Sperry, 1989; Choat *et al*., 2018), with P_50_, or the water potential (Ψ_l_) at which 50% of the xylem is embolized being a common metric for comparing drought tolerance between species (Brodribb, 2009; Choat *et al*., 2018).

Of the dozen studies that have assessed embolism resistance in herbaceous species (Nardini *et al*., 2008; Rosenthal *et al*., 2010; Lens *et al*., 2013, 2016; Nolf *et al*., 2014; Skelton *et al*., 2017; Zhang & Brodribb, 2017; Ahmad *et al*., 2018; Cardoso *et al*., 2018; Dória *et al*., 2018, 2019; Thonglim *et al*., 2021; Dong *et al*., 2022; Huber *et al*., 2023; Haverroth *et al*., 2024; Pereira *et al*., 2024; Rimer & McAdam, 2024), few studies have investigated a large number of species or attempted to associate phenology with embolism resistance. Most studies have focused on stem embolism resistance in herbs (Lens *et al*., 2016; Skelton *et al*., 2017; Dória *et al*., 2018, 2019), with few studies reporting leaf embolism resistance (Haverroth *et al*., 2024; Pereira *et al*., 2024; Rimer & McAdam, 2024). Within herbaceous species, increased embolism resistance as stems produce secondary xylem has been observed in *Solanum lycopersicum* L. (Solanaceae) and *Senecio minimus* Poir. (Asteraceae), being attributed to flowering and seed set (Haverroth *et al*., 2024). This development of more embolism resistant stem xylem results in vulnerability segmentation between the basal secondary xylem and the cauline primary xylem (Haverroth *et al*., 2024). There are no reports of vulnerability segmentation across organs in herbaceous species that lack secondary xylem (Skelton *et al*., 2017; Haverroth *et al*., 2024; Rimer & McAdam, 2024). Some herbaceous species have evolved increased embolism resistance as an adaptation to arid environments, contributing to the evolution of insular woodiness to survive more xeric habitats (Lens *et al*., 2013). This adaptation is evident in closely related Brassicaceae and Asteraceae species on oceanic islands, where embolism resistance increases with decreasing mean annual precipitation (Dória *et al*., 2019) and is further supported by a documented 1 MPa variation in stem P_50_ among populations of *Solidago canadensis* L. (Asteraceae) growing in dry vs humid sites in Austria (Nolf *et al*., 2014). These results suggest that hydraulic and anatomical traits in herbaceous species can rapidly acclimate to water availability, highlighting the crucial role of adaptation in embolism resistance with herbaceous species ecology.

Water availability rarely compromises growth in species that flower in the spring when soil water tables are highest, and drought is rare (Kemp, 1983; Lapointe, 2001). By contrast, species that flower in the late summer often experience periods of lower water availability during reproduction (Hayhoe *et al*., 2007), including episodes of summer drought before flowering (Misson *et al*., 2011). This temporal divergence in water availability suggests that hydraulic traits in spring and late‐summer‐flowering species may be under distinct selective pressures. Cardoso *et al*. (2018) reported coordinated plasticity between leaf osmotic potential and embolism resistance in *Helianthus annuus* L. (Asteraceae) plants when grown under well‐watered and water‐limiting conditions, suggesting herbaceous species may exhibit seasonal plasticity in embolism resistance and the Ψ_l_ at which bulk turgor is lost (TLP). TLP is a critical functional trait because it associates with the Ψ_l_ at which stomata close (Brodribb & Holbrook, 2003; Kane & McAdam, 2023), likely because the hormone abscisic acid, which mediates stomatal closure, is synthesized at TLP (Pierce & Raschke, 1980; McAdam & Brodribb, 2016; Bacete *et al*., 2021). Stomatal closure during drought is essential for preventing further decreases in Ψ_l_, thus delaying embolism, and the point of stomatal closure a critical trait to compare drought strategies.

While embolism resistance determines the survival limit of a plant under terminal drought, and TLP determines when the stomata will close under drought, these two traits together can be used to determine stomatal safety margin (SSM), which is the Ψ_l_ difference between when stomata close (or TLP) during drought and when significant embolism forms in the xylem (P_50_) (Chen *et al*., 2022). Large SSM are found in species that close stomata during drought well before embolism forms in the xylem, adopting a hydraulically safe strategy (Meinzer *et al*., 2009; Chen *et al*., 2019; Petek‐Petrik *et al*., 2023). By contrast, species with narrow SSM, allow Ψ_l_ to approach or even overlap with P_50_ before stomata begin to close. Species with a small SSM are thought of as prioritizing carbon acquisition and thus risk embolism formation during drought. Increasing evidence suggests that there is variation in both embolism resistance and SSM, which is primarily driven by edaphic gradients and environment, reflecting crucial tradeoffs between hydraulic safety and carbon gain (Johnson *et al*., 2012; Delzon & Cochard, 2014). These findings underscore a key ecological trade‐off between hydraulic safety and carbon gain. Similarly, leaf mass per unit area (LMA) is a key integrative trait reflecting the structural investment in leaf tissues and is often associated with drought tolerance (Poorter *et al*., 2009), with higher LMA typically linked to greater resistance to hydraulic failure, while species with lower LMA tending to prioritize rapid resource acquisition and shorter leaf lifespans (De La Riva *et al*., 2016). Among herbaceous species, annuals typically exhibit lower LMA than perennials, aligning with fast vs slow life‐history strategies (Garnier & Laurent, 1994; Poorter *et al*., 2009). How LMA contributes to drought tolerance in phenological distinct herbaceous species remains poorly studied.

Here, we assessed leaf embolism resistance in 41 temporally disjunct (separated by seasons), herbaceous species native to the central forest‐grassland ecotone of North America, categorized as either spring‐flowering (fruiting before the summer solstice) or late‐summer‐flowering (flowering until after the autumnal equinox). We chose to measure leaf embolism resistance because the hydraulic failure of leaves by embolism correlates with unrecoverable declines in leaf productivity during drought (Scoffoni *et al*., 2017; Cardoso *et al*., 2020; Brodribb *et al*., 2021). We sought to investigate whether there is an association between phenology and key ecophysiological traits including leaf P_50_, LMA, TLP, and SSM.

We hypothesized that late‐summer‐flowering species would exhibit greater leaf embolism resistance, larger SSM, and a greater LMA largely influenced by the need to survive periods of water limitation before and during flowering, when spring‐flowering species were dormant. We also tracked leaf embolism resistance monthly from early spring to leaf senescence in late‐summer in the perennial flowering herbaceous species *S. canadensis* to determine the degree of seasonally driven phenotypic plasticity in leaf embolism resistance in this species. *S. canadensis* is reported to have population‐level differences in leaf embolism resistance associated with recent adaptation to water availability (Nolf *et al*., 2014), suggesting either rapid local adaptation to aridity or strong phenotypic plasticity in this trait, making this species the perfect candidate to explore if it can also acclimate to water availability between seasons.

## Materials and Methods

### Sample selection

**Fig. 1:**
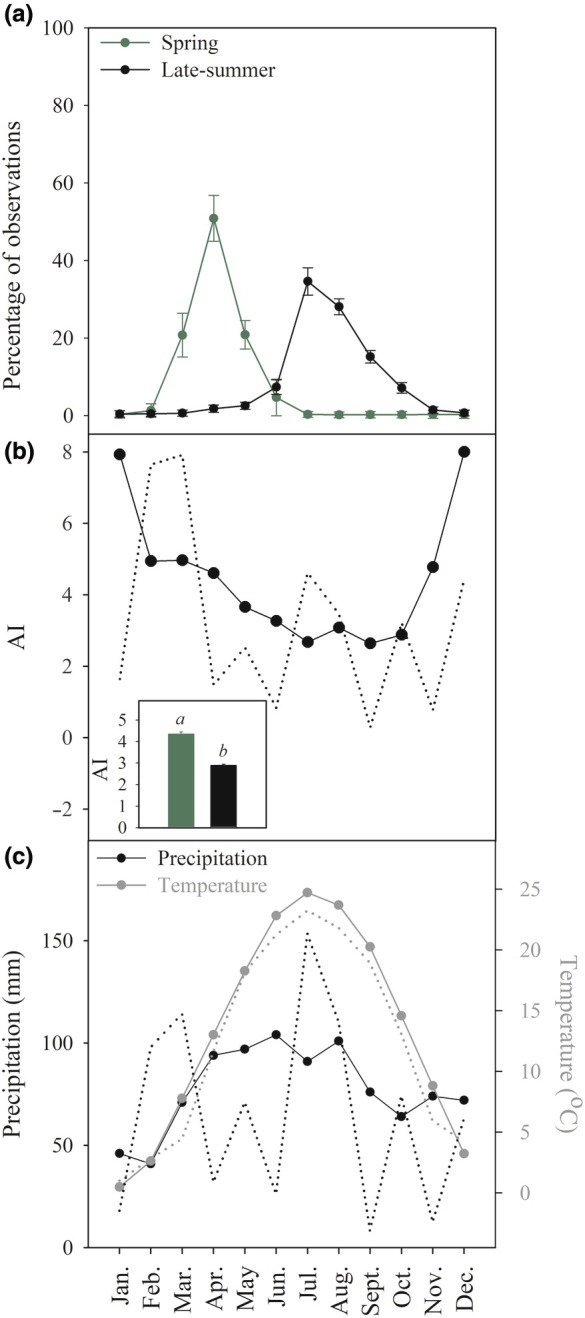
Contrasting growing seasons and climates of spring‐ and late‐summer‐flowering communities. (a) The mean (± SE) monthly normalized observations of species taken from publicly available datasets (https://www.inaturalist.org/) for the spring (green) and late‐summer‐(black)‐flowering species. Observations of flowers only of species were constrained to Central Indiana. Data for each individual species is shown in Supporting Information Fig. S1. (b) Mean monthly aridity index (AI). The corresponding AI for the study year (2023) is represented by the black dotted line. The insert in (b) depicts the average AI for each corresponding flowering time. Significant differences in AI between the flowering times are denoted with italicized letters (*t*‐test) between spring (green) and late‐summer (black)‐flowering native herbaceous species. (c) Mean monthly precipitation (mm) (black line) and temperature (°C) (dark gray line) of West Lafayette, Tippecanoe Co., IN 1970–2000 with a spatial resolution of 1 km^2^ from data extracted from WorldClim. The corresponding precipitation (mm) (dotted black line) and temperature (°C) (dotted dark grey line) for the study year (2023) of West Lafayette, Tippecanoe Co., IN.

In 2023, species were collected from abutting forest and prairie communities on Purdue University properties in and around Tippecanoe County, IN (Table 1). Spring‐flowering species were classed as those that flowered before the summer solstice (Fig. 1a), and in all species in this guild measurements were made on leaves had expanded in full sun before the canopy closed. In contrast, late‐summer‐flowering species were classed as species that flowered after the autumnal equinox (Fig. 1a), and all measured individuals grew in full sun, with forest understory species that were late‐summer‐flowering sampled from forest edges. INaturalist data from Central Indiana, using only the research grade flower observations, were used to determine respective flowering times for each species (Supporting Information Fig. S1). The percentage of monthly observations for each species was normalized by the total number of monthly observations. With the normalized percentage values, monthly means were made across all species in each respective flowering time (Figs 1a, S1). Species in each of the corresponding phenological classes (spring or late‐summer‐flowering) experienced contrasting climatic conditions and water availability during peak growing months (Fig. 1b,c). In all species, all measurements were taken during each species peak flowering time. Spring‐flowering species were collected from the end of February until the first week of May, whereas late‐summer‐flowering species were collected from the beginning of August until the beginning of October. Only plants that exhibited no herbivory or diseases were selected for assessment. Maximum plant height data for each species was taken from Britton & Brown (1970).

**Table 1:** The mean P_50_ (*n* = 3, ±SD), osmotic potential at full turgor (OP; *n* = 9, ±SD), turgor loss point (TLP), stomatal safety margin (SSM; *n* = 3, ±SD), and leaf mass per unit area (LMA; *n* = 5, ±SD) of 40 native Indiana herbaceous species with the corresponding species, families, and life cycle.

Family	Species	Season	Ecological guild	Habitat	Maximum plant height (cm)	P_50_ (MPa)	OP (MPa)	TLP (MPa)	SSM (MPa)	LMA (g m^−2^)
Apiaceae	*Erigenia bulbosa* Nutt.	Spring	Perennial geophyte	Forest	22.86	−2.02 ± 0.06	−0.99 ± 0.03	−1.46 ± 0.03	0.57 ± 0.07	28 ± 9.0
Apiaceae	*Zizia aurea* (L.) W.D.J.Koch	Spring	Perennial	Forest	76.2	−2.06 ± 0.39	−0.67 ± 0.03	−1.19 ± 0.03	0.88 ± 0.38	45 ± 9.0
Asteraceae	*Antennaria plantaginifolia* (L.) Richardson	Spring	Perennial	Prairie	6.09	−1.02 ± 0.19	−1.06 ± 0.03	−1.51 ± 0.03	−0.49 ± 0.20	52 ± 6.0
Asteraceae	*Erigeron philadelphicus* L.	Spring	Perennial	Prairie	91.44	−2.12 ± 0.14	−1.81 ± 0.02	−2.14 ± 0.02	−0.01 ± 0.15	61 ± 18.0
Asteraceae	*Packera glabella* (Poir.) C.Jeffrey	Spring	Annual	Prairie	91.44	−3.87 ± 0.63	−2.40 ± 0.04	−2.63 ± 0.04	1.23 ± 0.63	35 ± 3.0
Berberidaceae	*Caulophyllum thalictroides* (L.) Michx.	Spring	Perennial	Forest	91.44	−2.43 ± 0.24	−1.71 ± 0.04	−2.05 ± 0.04	0.38 ± 0.24	31 ± 3.0
Boraginaceae	*Mertensia virginica* (L.) Pers. ex Link	Spring	Perennial	Forest	60.96	−2.94 ± 0.08	−0.99 ± 0.02	−1.46 ± 0.03	1.48 ± 0.09	34 ± 3.0
Boraginaceae	*Hydrophyllum appendiculatum* Michx.	Spring	Biennial	Forest	60.96	−1.84 ± 0.38	−1.01 ± 0.03	−1.47 ± 0.03	0.37 ± 0.39	39 ± 6.0
Brassicaceae	*Cardamine concatenata* (Michx.) O.Schwarz	Spring	Perennial geophyte	Forest	38.1	−2.48 ± 0.48	−1.48 ± 0.04	−1.87 ± 0.04	0.62 ± 0.49	52 ± 3.0
Caprifoliaceae	*Valerianella umbilicata* (Sull. ex A.Gray) Alph.Wood	Spring	Annual	Forest	45.72	−0.93 ± 0.11	−0.73 ± 0.02	−1.24 ± 0.02	−0.30 ± 0.11	27 ± 6.0
Geraniaceae	*Geranium maculatum* L.	Spring	Perennial	Forest	60.96	−1.78 ± 0.09	−0.91 ± 0.02	−1.39 ± 0.02	0.39 ± 0.10	36 ± 3.0
Limnanthaceae	*Floerkea proserpinacoides* Willd.	Spring	Annual	Forest	38.1	−1.07 ± 0.30	−0.56 ± 0.03	−1.10 ± 0.03	−0.03 ± 0.30	17 ± 15.0
Montiaceae	*Claytonia virginica* L.	Spring	Perennial geophyte	Forest	30.48	−1.45 ± 0.19	−0.69 ± 0.03	−1.21 ± 0.03	0.24 ± 0.20	34 ± 6.0
Orobanchaceae	*Pedicularis canadensis* L.	Spring	Perennial	Forest	45.72	−1.27 ± 0.34	−0.63 ± 0.03	−1.16 ± 0.03	0.11 ± 0.34	41 ± 3.0
Papaveraceae	*Dicentra cucullaria* (L.) Bernh.	Spring	Perennial	Forest	25.4	−1.13 ± 0.62	−0.72 ± 0.04	−1.23 ± 0.04	−0.10 ± 0.62	28 ± 6.0
Plantaginaceae	*Collinsia verna* Nutt.	Spring	Annual	Forest	60.96	−1.83 ± 0.51	−1.26 ± 0.03	−1.68 ± 0.03	0.15 ± 0.50	22 ± 9.0
Polemoniaceae	*Phlox divaricata* L.	Spring	Perennial geophyte	Forest	60.96	−1.86 ± 0.13	−1.27 ± 0.03	−1.69 ± 0.03	0.17 ± 0.13	36 ± 12.0
Ranunculaceae	*Ranunculus abortivus* L.	Spring	Perennial	Forest	60.96	−3.00 ± 0.52	−2.00 ± 0.02	−2.29 ± 0.02	0.71 ± 0.51	30 ± 18.0
Rosaceae	*Geum vernum* (Raf.) Torr. & A.Gray	Spring	Perennial	Forest	60.96	−3.33 ± 0.34	−2.49 ± 0.04	−2.70 ± 0.04	0.63 ± 0.35	43 ± 3.0
Violaceae	*Viola pubescens* Aiton	Spring	Perennial	Forest	30.48	−1.81 ± 0.46	−1.28 ± 0.03	−1.69 ± 0.03	0.12 ± 0.46	32 ± 6.0
Amaranthaceae	*Amaranthus retroflexus* L.	Late‐summer	Annual	Prairie	304.8	−2.42 ± 0.94	−1.64 ± 0.09	−1.99 ± 0.07	0.43 ± 0.94	55 ± 9.0
Apiaceae	*Eryngium yuccifolium* Michx.	Late‐summer	Perennial geophyte	Prairie	182.88	−4.42 ± 2.51	−1.79 ± 0.06	−2.11 ± 0.05	2.31 ± 2.51	108 ± 9.0
Apiaceae	*Cicuta maculata* L.	Late‐summer	Perennial	Prairie	182.88	−1.35 ± 0.59	−1.27 ± 0.20	−1.69 ± 0.17	−0.34 ± 0.61	77 ± 21.0
Asteraceae	*Vernonia gigantea* (Walter) Trel.	Late‐summer	Perennial	Prairie	304.8	−2.19 ± 1.34	−1.38 ± 0.21	−1.78 ± 0.18	0.41 ± 1.36	83 ± 21.0
Asteraceae	*Erigeron strigosus* Muhl. Ex. Willd.	Late‐summer	Annual	Prairie	121.92	−3.28 ± 0.91	−1.55 ± 0.11	−1.92 ± 0.09	1.36 ± 0.92	50 ± 12.0
Asteraceae	*Rudbeckia triloba* L.	Late‐summer	Perennial	Prairie	152.4	−2.80 ± 1.20	−2.16 ± 0.02	−2.43 ± 0.02	0.37 ± 1.20	39 ± 12.0
Asteraceae	*Solidago canadensis* L.	Late‐summer	Perennial	Prairie	152.4	See Table 4				
Boraginaceae	*Hackelia virginiana* (L.) I.M.Johnst.	Late‐summer	Biennial	Forest	121.94	−2.74 ± 0.26	−1.62 ± 0.03	−1.97 ± 0.03	0.77 ± 0.26	53 ± 6.0
Campanulaceae	*Campanula americana* L.	Late‐summer	Annual	Forest	182.88	−3.76 ± 1.35	−0.95 ± 0.03	−1.42 ± 0.03	2.34 ± 1.35	29 ± 3.0
Fabaceae	*Senna hebecarpa* (Fernald) H.S.Irwin & Barneby	Late‐summer	Perennial	Prairie	182.88	−2.64 ± 0.78	−2.15 ± 0.03	−2.42 ± 0.02	0.22 ± 0.78	52 ± 26.0
Hypericaceae	*Hypericum aviculariifolium* Jaub. & Spach	Late‐summer	Perennial	Prairie	91.44	−2.60 ± 0.26	−1.87 ± 0.06	−2.19 ± 0.05	0.42 ± 0.27	34 ± 9.0
Lamiaceae	*Physostegia virginiana* (L.) Benth.	Late‐summer	Perennial	Prairie	121.92	−1.21 ± 0.04	−1.20 ± 0.15	−1.63 ± 0.12	−0.42 ± 0.12	70 ± 12.0
Malvaceae	*Sida spinosa* L.	Late‐summer	Annual	Prairie	60.96	−3.29 ± 0.69	−1.17 ± 0.03	−1.60 ± 0.02	1.69 ± 0.70	32 ± 6.0
Onagraceae	*Oenothera biennis* L.	Late‐summer	Biennial	Prairie	182.88	−2.86 ± 0.56	−1.21 ± 0.05	−1.64 ± 0.04	1.23 ± 0.57	78 ± 15.0
Orobanchaceae	*Aureolaria grandiflora* (Benth.) Pennell	Late‐summer	Perennial	Forest	91.44	−4.17 ± 1.05	−2.16 ± 0.02	−2.43 ± 0.02	1.74 ± 1.06	53 ± 3.0
Plantaginaceae	*Veronicastrum virginicum* (L.) Farw.	Late‐summer	Perennial	Prairie	213.36	−3.79 ± 0.85	−1.02 ± 0.15	−1.48 ± 0.12	2.31 ± 0.86	70 ± 15.0
Polemoniaceae	*Phlox paniculata* L.	Late‐summer	Perennial	Prairie	182.88	−1.85 ± 0.61	−0.86 ± 0.04	−1.35 ± 0.03	0.50 ± 0.61	78 ± 15.0
Polygonaceae	*Persicaria punctata* Small	Late‐summer	Annual	Forest	91.44	−2.22 ± 0.43	−1.19 ± 0.06	−1.62 ± 0.05	0.60 ± 0.43	59 ± 27.0
Rosaceae	*Agrimonia rostellata* Wallr.	Late‐summer	Perennial	Forest	182.88	−4.41 ± 0.37	−3.35 ± 0.31	−3.42 ± 0.26	0.99 ± 0.44	33 ± 6.0
Solanaceae	*Solanum carolinense* L.	Late‐summer	Perennial	Prairie	121.92	−1.79 ± 0.29	−1.41 ± 0.06	−1.80 ± 0.05	−0.01 ± 0.29	69 ± 21.0
Verbenaceae	*Verbena hastata* L.	Late‐summer	Perennial	Prairie	213.36	−2.74 ± 0.12	−1.61 ± 0.08	−1.97 ± 0.07	0.77 ± 0.14	75 ± 18.0

[Correction added on 8 July 2025, after first online publication: the Family detailed for the species *Senna hebecarpa* has been updated.]

### Climate data

Climate data for West Lafayette, IN were obtained from the WorldClim database (https://worldclim.com/), the data were extracted using the raster (v.3.6‐30; Hijmans, 2024) and ncdf4 (v.1.23) packages. Precipitation and temperature data were extracted based on the coordinates of Purdue University (40.42–86.93) for each month of the year based on historical climate data from 1970 to 2000 with a spatial resolution of 1 km^2^. The monthly aridity index (AI) was then calculated based on De Martonne (1926). Climate data for the study year (2023) was collected from Purdue Mesonet (https://ag.purdue.edu/indiana‐state‐climate/purdue‐mesonet/purdue‐mesonet‐data‐hub/).

### Sample collection for optical vulnerability analysis

Leaf vulnerability curves were performed using the optical vulnerability method and subsequent image analysis, outlined by Brodribb *et al*. (2016). Whole plants (three biological replicates per species) with the surrounding soil intact, to ensure minimal damage to the roots, were collected in the early morning, placed in a bucket of water, covered with a large opaque bag to prevent transpiration, and transported back to the laboratory. The soil was carefully washed away from the roots, and samples were allowed to rehydrate in a dish of water until Ψ_l_ had relaxed to > −0.1 MPa. At which point, whole plants then underwent a bench dehydration to determine leaf embolism resistance. The newest most fully expanded cauline leaf near or on the flowering stem was positioned on the stage of a trinocular stereomicroscope (SZM Series: AmScope: Irvine, CA, USA) equipped with a digital camera (MU163: AmScope). The leaf edges were taped to minimize noise from shrinkage or any movement during the drying process. Leaf images were recorded every 5 min during slow bench dehydration. Embolism events were identified from these image stacks as dark pixels directly overlaying the leaf veins due to the change in light transmission through the xylem caused by embolism. Ψ_l_ measurements were conducted using a Scholander Pressure Chamber (PMS International) every 2–3 h from leaves or leaflets neighboring the leaf being imaged, sampled during dehydration until the leaves on the plant were desiccated. A regression was then fitted against time and the Ψ_l_ values, which allowed for calculating the Ψ_l_ value for each image during dehydration. Subsequent image analysis of the leaf image stack was conducted as outlined in Brodribb *et al*. (2016). Embolism resistance was determined from image stacks of leaves that were then analyzed using imagej software following the instructions provided on GitHub (https://www.opensourceov.org/) the ‘OSOV’ toolbox was added to facilitate the image analysis process (https://github.com/OpenSourceOV/imagej‐scripts). Images were then individually analyzed for embolism events, as changes in light transmission through the xylem, so that at the end of image analysis, image stacks contained exclusively embolism events marked as dark pixels. An area of embolized xylem for each image stack was measured using the OSOV toolbox and then analyzed in Excel. The percentage of the embolized xylem was then calculated. A mean leaf vulnerability curve was constructed for each species following Cardoso *et al*. (2022), in which a mean value was calculated for each 5% increment in embolism formation.

### Assessment of seasonal hydraulic plasticity in *S. canadensis*

*Solidago canadensis* was used to track seasonal plasticity in leaf embolism resistance, TLP, LMA, and SSM in a monoculture population. In this species, measurements began on rosettes in spring (May), whereas the final measurements were made in the late summer (September), just before leaf senescence. This allowed us to assess if the late‐summer‐flowering species adapted to drier summer conditions by becoming more embolism resistant. In July, whole plants were used to test for variation in leaf embolism resistance across the canopy by measuring the leaf at the node bearing the most recent fully expanded leaf (cauline), the middle node, and the oldest node bearing a non‐senescent leaf.

### Turgor loss point and Stomatal Safety Margin

TLP for each species was determined from the relationship between the saturated osmotic potential at full turgor and TLP, according to Bartletta *et al*. (2016). Three biological replicate leaf samples were collected for each of the species from the same plants that were used to assess leaf embolism resistance. Samples were bagged to prevent transpiration and ensure the stomata were closed. Petioles from the three leaves were then cut under water and allowed to rehydrate for up to 3 h; once rehydrated (Ψ_l_ > −0.1 MPa), three‐hole punches were taken from each leaf and immediately frozen in an Eppendorf tube (Thermo Fisher Scientific, Waltham, MA, USA). Leaf disc samples were placed in a leaf psychrometer (ICT International, Armidale, NSW, Australia), sealed with petroleum jelly, and put in an insulated box to ensure thermal stability. Samples were allowed to stabilize in the insulated box for an hour before measurement of Ψ_l_ with measurements taken every 10 min until there was < 0.05 difference in measured Ψ_l_ values. Averages were made across the three technical replicates and then used to create an average from the three leaves. The difference between the estimated TLP value and leaf P_50_ was calculated to determine SSM.

### Leaf mass per unit area

Five leaves from each species were taken to determine LMA. Leaves were scanned with an Epson Scanner, and subsequent leaf area was determined using imagej software. After scanning, the leaves were dried at 70°C for 72 h. Dry leaf mass was then measured with an analytical balance.

### Phylogenetic analysis

To test if P_50_, LMA, TLP, SSM, and phenology (spring and late‐summer‐flowering) were phylogenetically conserved or if differences in these key physiological traits are due to climatic differences between the seasons, we generated a phylogeny for the study species that was trimmed from Janssens *et al*. (2020) as the backbone of our tree. We assigned calibrated branch lengths for Orobanchaceae from Xu *et al*. (2022), Rosaceae from Zhang *et al*. (2017), Plantaginaceae from Wolfe *et al*. (2021), Boraginaceae from Mansion *et al*. (2009), Apiaceae from Wen *et al*. (2021), Asteraceae from Mandel *et al*. (2019) and Park & Potter (2015), and *Phlox* from Rose (2018).

### Data analysis and statistics

ANOVAs and phylogenetic analysis were conducted with R v.4.3.1. (R Core Team, 2025). To test if P_50_ and phenology (spring and late‐summer‐flowering) were phylogenetically conserved, we used phytools (v.2.3.0; Revell, 2025), caper (v.1.0.3; Orme, 2023), and ape (v.5.8; Paradis & Schliep, 2019) R packages. We tested for a phylogenetic signal in P_50_, TLP, SSM, and LMA and phenology (spring or late‐summer flowering) by calculating Blomberg's *K* (O'Meara, 2012; Revell & Harmon, 2022) and Pagels λ (O'Meara, 2012; Revell & Harmon, 2022) for both P_50_ and phenology. We also performed a phylANOVA to test significant phylogenetic differences between the P_50_, TLP, SSM, and LMA of spring and late‐summer‐flowering species. Pairwise comparisons between all measured traits means were made with the Tukey HSD test between Ψ_l_ corresponding to P_12_, P_50_ and P_88_ for each species. The multcompview package (v.1.4‐26; Graves & Piepho, 2024) was used to denote letters indicating significant differences between species in each flowering time.

## Results

### Variation in leaf embolism resistance between spring‐ and late‐summer‐flowering species

**Fig. 2:**
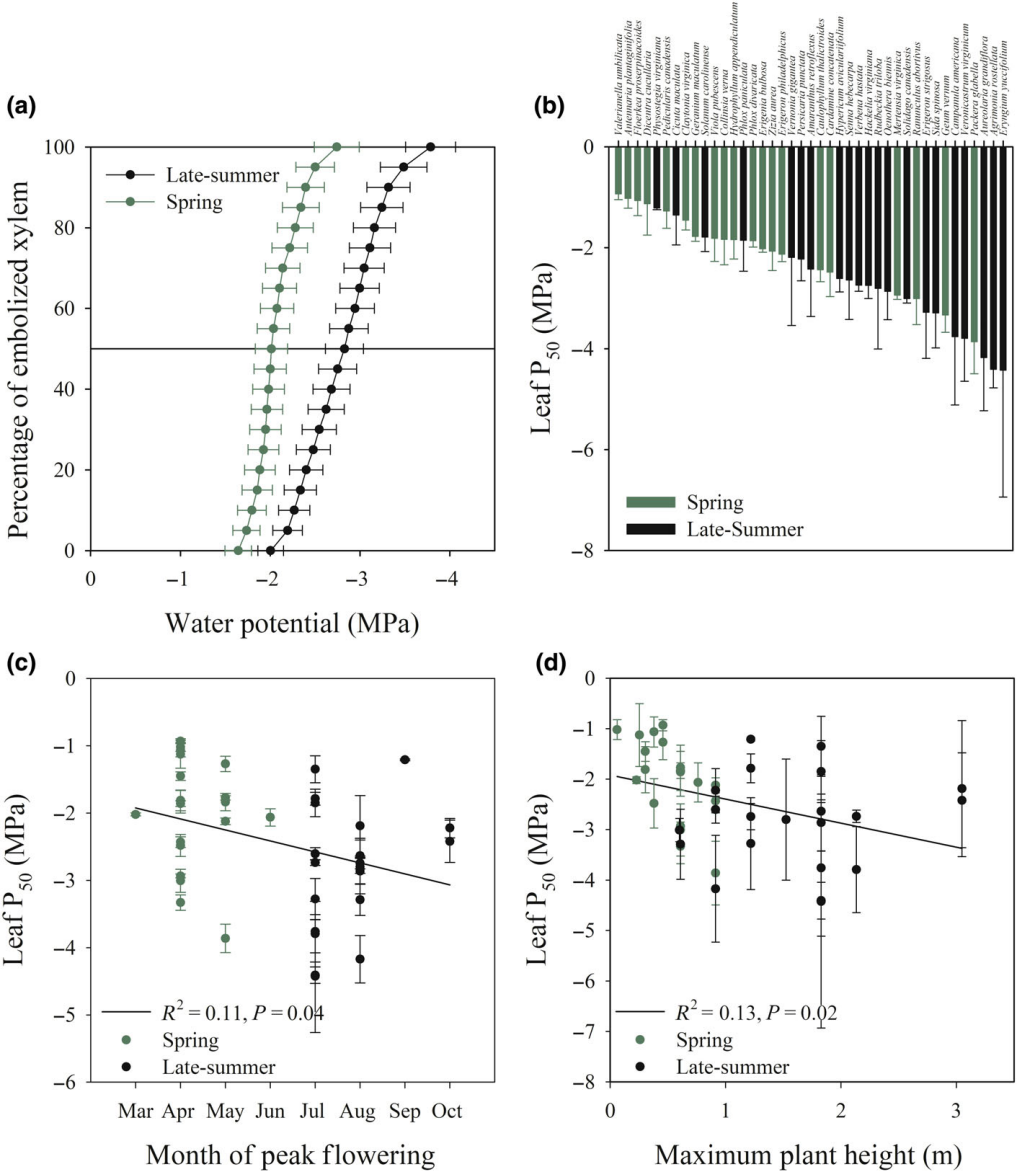
Late‐summer‐flowering herbaceous species tend to have more embolism‐resistant leaf xylem. (a) Mean leaf optical vulnerability curves (*n* = 20, mean ± SE) of the 20 species that flower before the summer solstice and the 20 species that flower before the autumnal equinox. Late‐summer‐flowering species are denoted with black lines and dots, whereas spring‐flowering species are denoted with a green line and dots. The solid horizontal line represents 50% of the embolized xylem area. (b) Mean leaf P_50_ (*n* = 3, mean ± SD) of all 41 species surveyed; spring species are denoted with a green box, and late‐summer species are denoted with a black box. (c) Relationship of mean leaf P_50_ and month of peak flowering. (d) Relationship between leaf P_50_ and maximum plant height (m). The leaf P_50_ and maximum height values of all species can be found in Supporting Information Table 1.

**Fig. 3:**
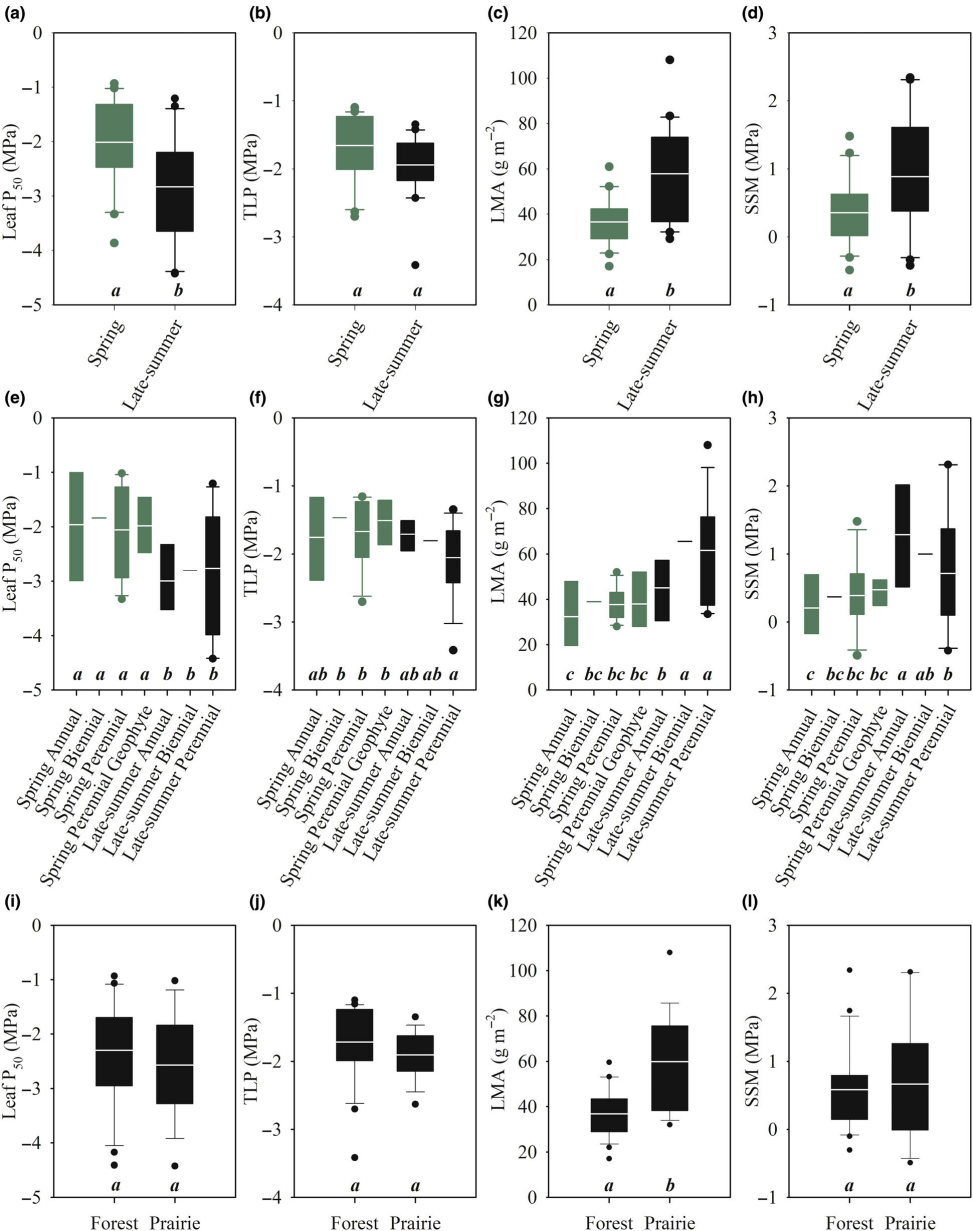
Seasonal timing, life history, and habitat influence key hydraulic and structural traits in herbaceous species. Box and whisker plots showing the 25 and 75 quartile range with the mean depicted as a white line through the box for (a) leaf P_50_ (MPa), (b) turgor loss point (TLP), (c) leaf mass per unit area (LMA), and (d) stomatal safety margin (SSM) between spring (green) and late‐summer‐flowering (black) species. Partitioning of mean (e) leaf P_50_, (f) TLP, (g) LMA, (h) SSM based on ecological guild is shown; and mean (i) leaf P_50_, (j) TLP, (k) LMA, (l) SSM based on species habitat preference. Significant differences in means are denoted by different letters at the bottom of the panels.

Significant differences in AI between spring and late‐summer (*P* < 0.05, *t*‐test) occur in West Lafayette, IN, with an average AI of the spring of 4.34 ± 0.10 (*n* = 3 months, ±SE; Fig. 1b) and late‐summer of 2.90 ± 0.05 (*n* = 3 months, ±SE; Fig. 1b). We found no significant differences in temperature (*t*.test = 0.05), precipitation (*t*.test = 0.40), or AI (*t*.test = 0.18) between the collected WorldClim data (1970–2000) and the study year (2023). Mean leaf P_50_ was less negative in species that flowered in the spring compared to late‐summer (Fig. 2a; Table 2, ANOVA). The mean leaf P_50_ for the spring‐flowering species was −2.01 ± 0.18 MPa (Fig. 2a; Table 2), whereas the mean leaf P_50_ for the late‐summer‐flowering species was −2.83 ± 0.21 MPa (Fig. 2a; Table 2). There was considerable variation in leaf embolism resistance across species (Figs S2 and S3); the most vulnerable leaf xylem was measured in the spring‐flowering species *Valerianella umbilicata* (Sull. ex A.Gray) Alph.Wood (Caprifoliaceae) with a mean P_50_ of −0.93 ± 0.11 MPa (Fig. 2b; Table 1), while the most embolism resistant species was measured in the late‐summer‐flowering species *Eryngium yuccifolium* Michx. (Apiaceae) with a mean P_50_ of −4.42 ± 2.51 MPa (Fig. 2b; Table 1). We found a significant relationship between mean leaf P_50_ and the month of peak flowering (Fig. 2c), with species that experienced peak flowering earlier in the year having a significantly less negative leaf P_50_ than species that flowered later in the year (Fig. 2c). We also found a significant relationship between maximum plant height and mean leaf P_50_ (Fig. 2d), with taller plants having a more negative leaf P_50_ than shorter plants. Mean P_50_ of spring and late‐summer‐flowering species did not differ based on ecological guild (annual, biennial, perennial, or geophyte) or habitat preference (prairie vs forest) (Fig. 3e,i).

**Table 2:** The mean P_50_, OP, turgor loss point (TLP), stomatal safety margin (SSM), and leaf mass per unit area (LMA) (*n* = 20 in spring and 21 in late‐summer, ± SE) herbaceous species for each of the corresponding flowering times.

Season	P_50_ (MPa)	OP (MPa)	TLP (MPa)	SSM (MPa)	LMA (g m^−2^)
Spring	−2.01 ± 0.18	−1.24 ± 0.13	−1.66 ± 0.11	0.35 ± 0.11	37 ± 2.3
Late‐summer	−2.83 ± 0.21	−1.57 ± 0.12	−1.94 ± 0.10	0.88 ± 0.19	58 ± 4.7

### Ecological trends in leaf turgor relations, SSM, and structure

**Fig. 4:**
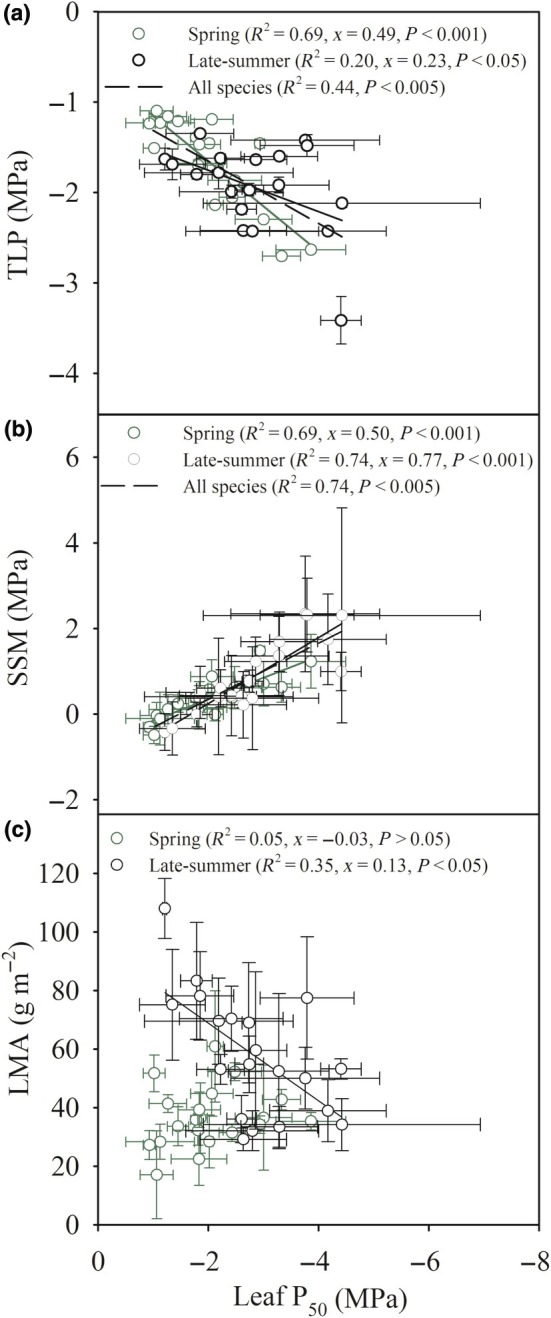
Leaf embolism resistance was associated with turgor loss point in spring‐flowering species and most strongly with leaf mass per unit area (LMA) in late‐summer‐flowering species, while stomatal safety margin (SSM) was driven by embolism resistance in all species. (a) The relationship between the Ψ_l_ at which 50% of the leaf xylem was embolized (leaf P_50_) and turgor loss point (TLP) for spring‐flowering species (green) and late‐summer‐flowering species (black) (*n* = 3 per species, mean ± SD). The relationship across all species measured is depicted as a black dashed line. (b) The relationship between leaf P_50_ and SSM for spring‐flowering species (green) and late‐summer‐flowering species (black) (*n* = 3 per species, mean ± SD). The relationship across all species is depicted as a black dashed line. (c) The relationship between leaf P_50_ and LMA for spring‐flowering species and late‐summer‐flowering species (*n* = 3 per species, mean ± SD). *R* ^2^ value, and corresponding slope (*x*), and significance values are shown for all significant linear regressions for spring and late‐summer species. Mean and SD values for each trait in all species can be found in Table 1.

We found that the spring‐flowering species had an average TLP of −1.66 ± 0.11 MPa (*n* = 20, ± SE; Table 2), and the late‐summer‐flowering species had an average TLP of −1.94 ± 0.11 MPa (*n* = 20, ±SE; Table 2), but this difference was not significant (Fig. 3b, *P* > 0.05). Late‐summer‐flowering perennial species had a lower TLP than spring‐flowering non‐annuals (Fig. 3f; Table 3). There was no significant difference in TLP between forest and prairie species (Fig. 3j). We observed a significant linear relationship between leaf P_50_ and TLP across all species (*R* ^2^_total_ = 0.44, *P* < 0.005, Fig. 4a), including within the sampled spring‐flowering species (*R* ^2^_spring_ = 0.69, *x* = 0.49, *P* < 0.001; Fig. 4a), and a weaker relationship within the sampled late‐summer‐flowering species (*R* ^2^_late‐summer_ = 0.20, *x* = 0.23, *P* < 0.05; Fig. 4a). Across all species measured, TLP varied by 1.83 MPa.

**Table 3:** The mean P_50_, osmotic potential at full turgor (OP), turgor loss point (TLP), stomatal safety margin (SSM), and leaf mass per unit area (LMA) (*n*, ± SE) of the 41 native Indiana herbaceous species for each of the corresponding flowering times separated by spring and late summer perennials, annuals, biennials, and geophytes.

Season	Leaf P_50_ (MPa)	OP (MPa)	TLP (MPa)	SSM (MPa)	LMA (g m^−2^)
Spring Perennial	−2.06 ± 0.24	−1.25 ± 0.18	−1.67 ± 0.15	0.39 ± 0.16	37.73 ± 2.11
Spring Annual	−1.96 ± 0.53	−1.35 ± 0.34	−1.76 ± 0.28	0.21 ± 0.27	32.4 ± 7.74
Spring Biennial	−1.84 ± 0.12	−1.01 ± 0.02	−1.47 ± 0.02	0.37 ± 0.15	39.00 ± 2.1
Spring Perennial Geophyte	−1.99 ± 0.3	−1.06 ± 0.23	−1.51 ± 0.19	0.47 ± 0.12	38.00 ± 7.21
Late‐summer Perennial	−2.77 ± 0.31	−1.71 ± 0.18	−2.05 ± 0.15	0.71 ± 0.25	61.6 ± 6.27
Late‐summer Annual	−3.00 ± 0.29	−1.30 ± 0.13	−1.71 ± 0.11	1.28 ± 0.35	45.08 ± 6.13
Late‐summer Biennial	−2.8 ± 0.06	−1.41 ± 0.2	−1.81 ± 0.17	1.00 ± 0.23	65.59 ± 12.56

The average SSM for sampled spring‐flowering species was 0.35 ± 0.11 MPa (*n* = 20, ±SE; Fig. 3d; Table 2) and 0.88 ± 0.18 MPa (*n* = 20, ±SE; Fig. 3d; Table 2) for the sampled late‐summer‐flowering species (Fig. 3d; Table 2), which was significantly different (Fig. 3h, *P* < 0.05). We found considerable variation in SSM across all species sampled (Fig. 4b; Table 1). Late‐summer‐flowering annual herbaceous species had larger SSM than all spring‐flowering species and late‐summer‐flowering perennials (Fig. 3h; Table 3). There was no significant difference in SSM between forest or prairie species (Fig. 3l). Across the sampled spring‐flowering species, SSM spanned 1.97 MPa; across the sampled late‐summer‐flowering species, SSM spanned 2.76 MPa. We found a significant relationship between leaf P_50_ and SSM across all species and when analyzed based on flowering time (*R* ^2^_spring_ = 0.69, *x* = 0.50, *P* < 0.001, *R* ^2^_late‐summer_ = 0.74, *x* = 0.77, *P* < 0.001, and *R* ^2^_total_ = 0.74; Fig. 4b). We did not find a relationship between SSM and TLP.

We found significant differences in LMA between species that flowered in the spring and late‐summer (Fig. 3c, *P* < 0.05, *t*‐test). The mean LMA for the spring‐flowering species was 37 ± 2.0 g m^−2^ (Fig. 3c; Table 2) and 58 ± 5.0 g m^−2^ in the late‐summer‐flowering species (Fig. 3c; Table 2). In all ecological guilds, late‐summer‐flowering biennial and perennial species had higher LMA than spring‐flowering counterparts (Fig. 3g; Table 3). Prairie species had a significantly larger LMA than all forest species (Fig. 3k). LMA did not correlate with leaf P_50_ across all species when taken together (Table S1). We also found no relationship between P_50_ and LMA among the spring‐flowering species (*R* ^2^_spring_ = 0.05, *x* = 0.03, *P* > 0.05; Fig. 4c; Table S2). There was a weak relationship between P_50_ and LMA among the late‐summer‐flowering species (*R* ^2^_late‐summer_ = 0.35, *x* = 0.13, *P* < 0.05; Fig. 4c; Table S3), with species with a low LMA having a more negative P_50_.

### No seasonal plasticity in ecophysiological traits in *S. canadensis*

**Fig. 5:**
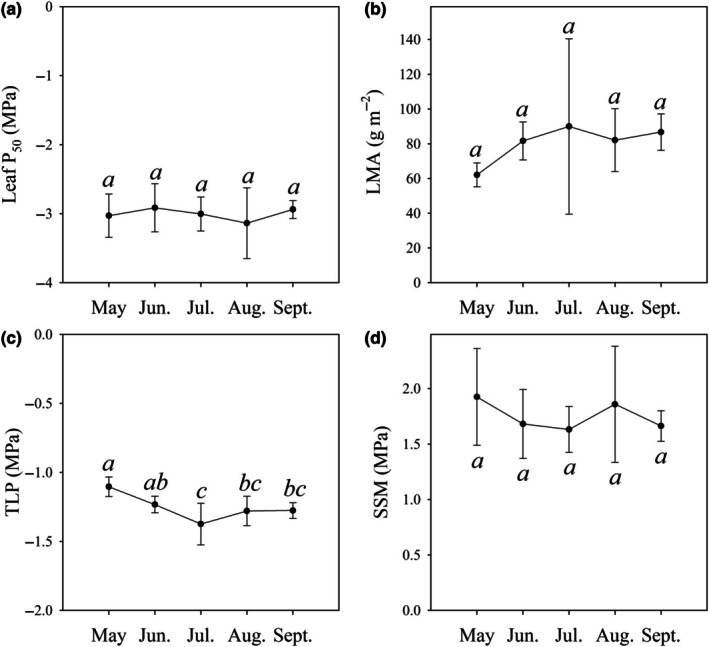
No change in key physiological functional traits across the season in the perennial, late‐summer‐flowering herbaceous species *Solidago canadensis*. (a) The mean Ψ_l_ at which 50% of xylem is embolized (P_50_, *n* = 3 ± SD), (b) leaf mass per unit area (LMA, *n* = 5 ± SD), (c) turgor loss point (TLP, *n* = 9 ± SD), and (d) stomatal safety margin (SSM, *n* = 3 ± SD) in plants of *S. canadensis* taken from a monoculture stand that was sampled on the first of each corresponding month. Significant differences between each month are denoted by different letters (ANOVA). Mean values for each of the points can be found in Table 4.

Mean leaf P_50_ of *S. canadensis* did not vary across the growing season (Fig. 5a), with minimal variation in LMA, and SSM in *S. canadensis* across the growing season (Fig. 5b,d; Table 4). Slight variation in TLP across the season was observed with a range of 0.27 between May having the least negative TLP of −1.10 ± 0.07 MPa, and July having the most negative TLP of −1.37 ± 0.15 MPa (Fig. 5c; Table 4). No significant differences in leaf P_50_ across the canopy of *S. canadensis* were observed when measured in July, further suggesting limited seasonal plasticity (Table 5; Fig. S4).

**Table 4:** Mean P_12_, P_50_, P_88_ (*n* = 3 ± SD), turgor loss point (TLP) (*n* = 9 ± SD), stomatal safety margin (SSM), and leaf mass per unit area (LMA) (g m^−2^) (*n* = 5 ± SD) of *Solidago canadensis* throughout a growing season between May and September.

Month	P_12_ (MPa)	P_50_ (MPa)	P_88_ (MPa)	TLP (MPa)	SSM (MPa)	LMA (g m^−2^)
May	−2.39 ± 0.89	−3.03 ± 0.31	−3.91 ± 0.67	−1.10 ± 0.07	1.93 ± 0.32	62 ± 6.84
June	−2.24 ± 0.88	−2.91 ± 0.35	−4.00 ± 0.56	−1.23 ± 0.06	1.68 ± 0.35	82 ± 10.94
July	−2.70 ± 0.69	−3.01 ± 0.23	−3.14 ± 0.26	−1.37 ± 0.15	1.63 ± 0.29	90 ± 50.48
August	−2.86 ± 0.63	−3.14 ± 0.51	−3.89 ± 0.90	−1.28 ± 0.11	1.86 ± 0.52	82 ± 18.19
September	−1.95 ± 0.76	−2.94 ± 0.13	−3.32 ± 0.28	−1.28 ± 0.06	1.66 ± 0.14	87 ± 10.48

**Table 5:** Mean P_12_, P_50_, P_88_ MPa (*n* = 3 ± SE) *Solidago canadensis* of leaves from the bottom, middle, and top from three individuals to test for leaf plasticity in embolism resistance across a single individual.

Tissue	P_12_ (MPa)	P_50_ (MPa)	P_88_ (MPa)
Bottom	−2.91 ± 0.21	−3.02 ± 0.15	−3.12 ± 0.14
Middle	−3.11 ± 0.01	−3.48 ± 0.11	−3.77 ± 0.16
Top	−3.18 ± 0.11	−3.44 ± 0.26	−3.59 ± 0.31

### Phylogeny does not explain variation in ecophysiological traits

**Fig. 6:**
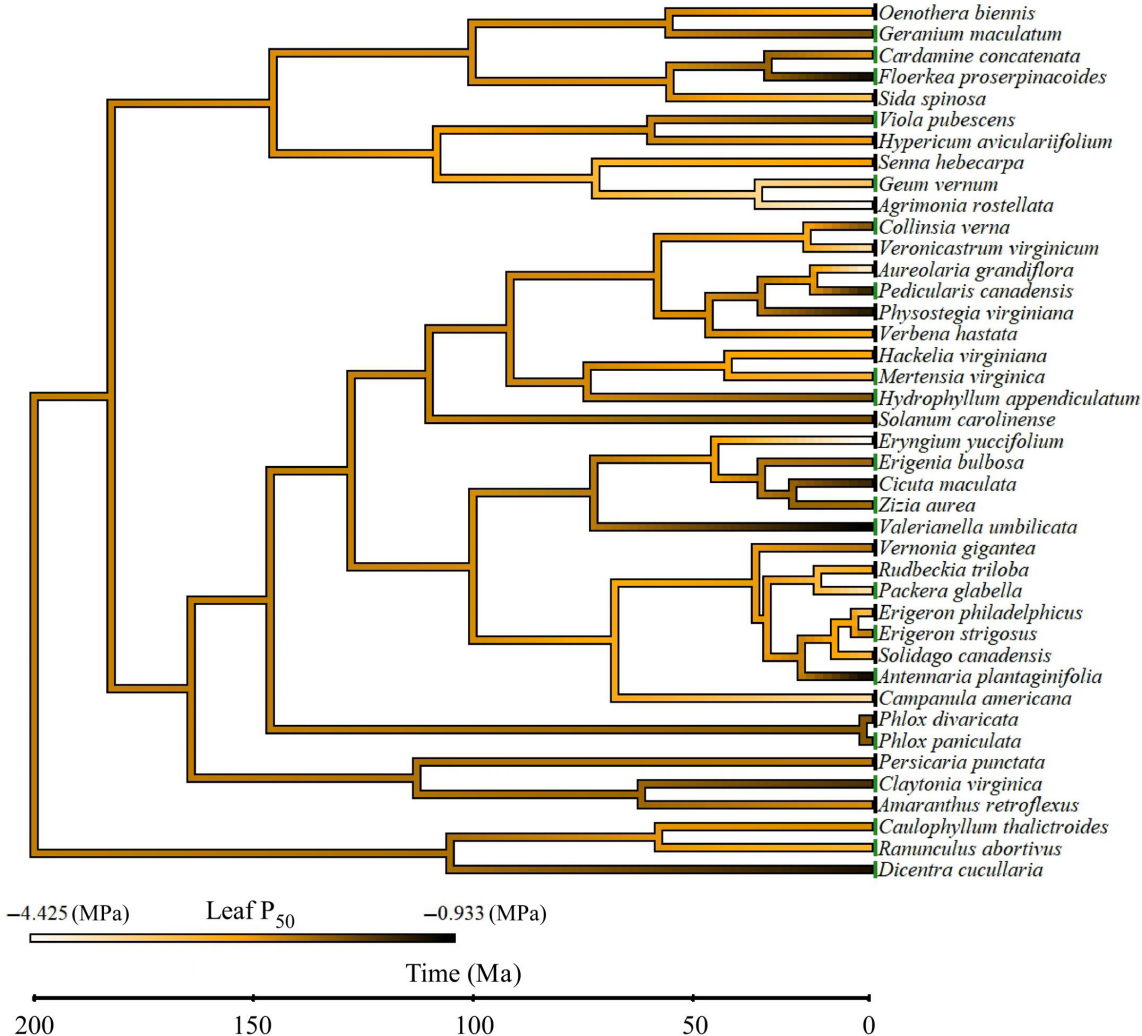
Ancestral state reconstruction of leaf embolism resistance in sampled, co‐occurring but temporally disjunct herbaceous angiosperms. Dated phylogeny of the 41 herbaceous species measured in this study with ancestral state reconstruction of the Ψ_l_ at which 50% of the leaf xylem has been embolized (leaf P_50_). Branches of the phylogeny are color‐coded according to leaf P_50_ (scale bottom left). The time scale on the bottom of the phylogeny has a root age of 190 Ma.

We found that eight of the 25 families in our analysis had species classified as spring‐ and late‐summer‐flowering, meaning that eight families had a spring‐flowering species and a separate late‐summer‐flowering species. While the remaining 17 families were represented by species that were constrained to flowering in only either spring or late‐summer, causing these 17 families to be either spring or late‐summer‐flowering families. Four families of the eight represented by species at both times of the year, namely Caprifoliaceae, Plantaginaceae, Orobanchaceae, and Rosaceae, had significant differences in mean leaf P_50_, LMA, TLP, and SSM between representative species that flowered in spring and late‐summer (Table 1). Late‐summer‐flowering species within these four families had a more negative leaf P_50_ and TLP as well as a larger LMA and SSM than spring‐flowering species (Table 1). By contrast, the two species of *Phlox* (Polemoniaceae), *P. paniculata* L. that flowered in spring and *P. divaricata* L. that flowered in the late summer, had similar leaf P_50_, LMA, TLP, and SSM (Table 1). The family with the greatest variation in mean P_50_ between species was Orobanchaceae, with mean leaf P_50_ spanning 2.9 MPa between the more vulnerable spring‐flowering species *Pedicularis canadensis* L. and the more embolism resistant late‐summer‐flowering species *Aureolaria grandiflora* (Benth.) Pennell (Table 1). Phylogenetic analysis (phylANOVA) confirmed a significant effect of phenology (either spring or late‐summer‐flowering) on leaf P_50_, with an *F*‐value of 9.28 and a corresponding *P*‐value of 0.002. The consistency of the ANOVA and the phylANOVA is supported by the lack of phylogenetic signal for leaf P_50_ across the 41 species sampled, with a Blomberg's *K*, of 0.708 and Pagels λ < 0.0001, suggesting no phylogenetic dependence on leaf P_50_ (Fig. 6). We found no phylogenetic signal in LMA, TLP, or SSM further suggesting no phylogenetic dependence on any of the measured traits (Table 6). Phylogenetic analysis of phenology as a binary trait revealed a similarly low Blomberg's *K* value of 0.435, indicating no phylogenetic conservatism in flowering time in the sampled species, suggesting that phenological classification was not biased by phylogeny.

**Table 6:** Phylogenetic results and Blomberg's *K* and Pagel's λ of all measured traits (P_50_, leaf mass per unit area (LMA), turgor loss point (TLP), and stomatal safety margin (SSM)) for all 41 herbaceous species measured here.

	Blomberg's *K*	Pagel's λ	*F* value	*P*‐value
P_50_	0.71	< 0.001	9.279	0.002
LMA	0.76	< 0.001	18.229	0.001
TLP	0.77	0.48	2.742	0.076
SSM	0.57	< 0.001	6.986	0.008

## Discussion

### Climatic variables drive variation in leaf embolism resistance across species

We found that leaf embolism resistance is associated with the phenology of temporally disjunct herbaceous species. Late‐summer‐flowering species, on average, exhibited more negative leaf P_50_ values, suggesting these species can survive to lower Ψ_l_ during the growing season than their spring‐flowering counterparts. Spring‐flowering species may have, on average, less embolism‐resistant leaf xylem because they rarely encounter severe soil water deficits and survive under conditions of a lower aridity. By contrast, late‐summer‐flowering species must tolerate summer drought, which is not uncommon, to reproduce (Ackerly, 2004; Volaire, 2008). The more vulnerable xylem in spring‐flowering species suggests that these species could be extremely sensitive to a spring drought (Cook *et al*., 2018; Anderson *et al*., 2020). These findings support the hypothesis that hydraulic strategies in herbaceous plants are shaped by distinct seasonal climatic pressures rather than phylogenetic constraint (Lens *et al*., 2016).

We found a significant difference in water availability (AI) (Fig. 1b,c) between the main flowering times of spring and late‐summer‐flowering species, which could explain differences in leaf P_50_. A number of the spring ephemeral species face unique hydraulic challenges due to the potential risks of embolism induced by late season frost events (Sperry & Sullivan, 1992; Lobos‐Catalán & Jiménez‐Castillo, 2023), which causes gas bubble formation in xylem conduits, that can expand upon thawing and cause damage to leaves (Sperry & Sullivan, 1992). It is hypothesized that there is a trade‐off between freezing and drought tolerance (Puglielli *et al*., 2021). The most vulnerable species in our dataset had peak flowering times in the earliest months of spring (Fig. 2c), when frosts are more likely. This suggests that there might be a trade‐off between freezing tolerance and embolism resistance in herbs.

The association between leaf P_50_ and phenology may also be due to spring ephemeral herbs adopting a short reproductive cycle and rapid growth rate to evade canopy shading (Forest *et al*., 1978; Rothstein & Zak, 2001). Spring‐flowering annual species had the lowest LMA (Fig. 3g), which supports the argument that there might be a trade‐off between growth rate, leaf life span, and leaf P_50_ in herbs (Garnier & Laurent, 1994; Poorter *et al*., 2009). We found a relationship between plant height and leaf P_50_; Givnish (1982) suggested that herbaceous plant height is due to intense competition for light with neighbors. Our results suggest that in herbaceous communities in which there is intense competition for light, there might also be intense competition for water driving increased embolism resistance. Heberling *et al*. (2019) showed that spring‐flowering perennials in deciduous forests undertake most carbon capture before canopy closure, emphasizing the importance of early season resource acquisition as a key ecological strategy. This supports our finding that spring annuals and ephemerals have a very narrow SSM, suggesting that these species prioritize carbon gain. The less embolism resistant leaf xylem of spring‐flowering species also highlights a potential vulnerability of these species to future earlier and more severe spring droughts under future climate scenarios (Swain & Hayhoe, 2015; Darenova *et al*., 2017; Martinuzzi *et al*., 2019).

In contrast to spring‐flowering species, many late‐summer‐flowering species, like *E. yuccifolium* and *Agrimonia rostellata* Wallr. (Rosaceae), adapted to open prairie or forest‐edge habitats, respectively, experience prolonged periods of reduced soil moisture and higher evaporative demand. These species have more embolism‐resistant leaves and larger SSM than most other herbaceous species (Figs 2b, 3d,h), which would ensure sustained hydraulic function and reproduction under conditions of periodic water limitation.

### Xylem vulnerability and stomatal regulation shape herbaceous species drought response

Some lineages like *Phlox* showed minimal differences in leaf P_50_ (even though both species grew in full sun) despite one species in this genus being spring‐flowering and the other one a late‐summer‐flowering species. This suggests other morphological or physiological traits may be driving differences in drought tolerance in this genus. These traits could include root architecture, leaf capacitance, as well as stomatal control, all of which serve as a buffer for declines in xylem Ψ during periods of drought (Koevoets *et al*., 2016; McCulloh *et al*., 2019; Haworth *et al*., 2021; Chen *et al*., 2022). Other traits not measured here that can confer increased drought resistance include increased leaf capacitance, lower minimum conductance, and deeper roots for enhanced ground water acquisition (Meinzer *et al*., 2009). Furthermore, carbohydrate storage can also contain osmotically active molecules that can be utilized to osmotically adjust to promote the maintenance of positive turgor during drought (Chapin *et al*., 1990; Sevanto, 2018; Lubbe *et al*., 2021). It is not known whether more vulnerable spring‐flowering species might utilize belowground carbohydrate and water storage to survive drought, which could facilitate survival in the absence of embolism resistant xylem.

We found no significant difference in TLP between the spring or late‐summer‐flowering species, suggesting that phenology alone may not account for the diversity in stomatal closure strategies among herbaceous angiosperms. The average TLP observed across the surveyed herbaceous species resembles values reported across woody species (Anderegg *et al*., 2016; Choat *et al*., 2018), and in native tree species in the same environment (Kannenberg *et al*., 2019). This indicates that despite differences in growth forms, herbaceous and woody angiosperms may converge on similar strategies for regulating stomatal closure relative to plant water status.

We found a strong correlation between leaf P_50_ and TLP across all species (Fig. 3a), suggesting that more embolism‐resistant species close stomata at a lower Ψ_l_ than more vulnerable species. Early stomatal closure to prevent embolism is common in xeric climates where plants must conserve water during the dry season (Bourne *et al*., 2017). Stomatal closure before the onset of embolism in the xylem is consistent with work in woody species (Delzon & Cochard, 2014; Bartletta *et al*., 2016), supporting a safety–efficiency trade‐off that governs (Henry *et al*., 2019) in plants (Henry *et al*., 2019). We found no correlation between SSM and TLP (*R* ^2^ = 0.04) but a strong correlation between SSM and leaf P_50_ (Fig. 3b). This suggests that in our sampled herbaceous flora leaf P_50_ rather than TLP primarily determines SSM. This differs somewhat from many woody species, where both TLP and leaf P_50_ can shape SSM (Brodribb *et al*., 2016; Martin‐StPaul *et al*., 2017, Guillemot *et al*., 2022).

### LMA correlates with leaf P_50_ among late‐summer‐flowering herbaceous species

In our study, late‐summer‐flowering herbaceous species had higher mean LMA than spring‐flowering species (Fig. 4c), and these differences also manifested within families. This pattern suggests that late‐summer‐flowering herbaceous species, which experience seasonally drier conditions, have greater leaf structural investment (Sporbert *et al*., 2022; Zhang *et al*., 2022). We found that late‐summer‐flowering non‐annuals have a greater LMA than spring and late‐summer annual counterparts (Fig. 3g). Despite this overall difference, we did not observe a relationship between LMA and leaf P_50_ among spring‐flowering species (Fig. 4c). Late‐summer‐flowering species showed a negative correlation between LMA and leaf P_50_, indicating that more embolism resistant individuals invested less in leaf mass (Fig. 4c). This finding contrasts with many prior reports of a positive LMA‐embolism resistance relationship in woody species (Nardini *et al*., 2012). High‐LMA leaves have greater construction costs and longevity, often correlating with higher resistance to hydraulic failure in low‐rainfall environments (Scholz *et al*., 2013; Nardini, 2022). Conversely, species from mesic habitats frequently have lower LMA and more vulnerable xylem (De La Riva *et al*., 2016). This suggests that herbaceous angiosperms can develop embolism resistant xylem in relatively low construction cost leaves, a leaf economics‐safety link that is rarely adopted by woody angiosperms.

### No evidence of seasonal plasticity in physiological traits in *S. canadensis*

Seasonally driven plasticity in leaf and stem embolism resistance has been reported in some woody and herbaceous angiosperms (Sorek *et al*., 2021, 2022; Ma *et al*., 2024); however, we found no within‐season shift in hydraulic traits due to water availability in *S. canadensis*. This species does show local adaptation in stem embolism resistance to water availability (Nolf *et al*., 2014), but we found that our sampled population does not adjust embolism resistance or other key physiological traits (TLP, LMA, or SSM) related to water use throughout a growing season (Fig. 5). Limited plasticity contrasts with reports from fast‐growing annuals like *Helianthus annuus*, which can shift osmotic traits in response to drought (Cardoso *et al*., 2018). This suggests that in *S. canadensis*, embolism resistance can adapt over short generational timescales to local water availability, as opposed to variation in embolism resistance between populations being a plastic response (Nolf *et al*., 2014). The lack of seasonal plasticity in *S. canadensis* may reflect a commitment to a hydraulically safe, late‐season reproduction strategy relying more on local adaptation to cope with environmental heterogeneity across seasons. The rapid adaptive potential of embolism resistance in this species (Nolf *et al*., 2014) may explain why no clear phylogenetic signal was detected in our analysis, with rapid evolution of leaf embolism resistance occurring in herbs.

### Leaf embolism resistance is an adaptive trait and shows little phylogenetic constraint among herbaceous angiosperms

We found that the range in leaf P_50_ in herbaceous angiosperm species is similar to the variation in leaf and stem P_50_ reported across tree species native to this region (Kannenberg *et al*., 2019; Rimer & McAdam, 2024). These findings align with broader evidence that leaf P_50_ in herbaceous plants can equal, or even exceed, that of co‐occurring woody species (Lens *et al*., 2016). Indeed, our phylogenetic analysis suggests that leaf P_50_ is a highly homoplastic trait within the herbaceous flora, with little if any phylogenetic signal (Fig. 6). While there is some evidence of a phylogenetic signal in phenology in herbaceous angiosperms (Sanchez‐Martinez *et al*., 2020; Gao *et al*., 2022; Ávila‐Lovera *et al*., 2023), the absence of a signal in our phylogenetic analysis of our sampled species indicates that the species sampled were randomly dispersed across the phylogeny of eudicots. Consequently, the weak phylogenetic signal we detected for leaf P_50_ implies that this trait is under strong selection driven by seasonal climatic variables during peak flowering. This conclusion is supported by prior work, which suggests that extreme embolism resistance has independently evolved numerous times in seed plants (McAdam & Cardoso, 2019). Although some hydraulic traits are often viewed as phylogenetically conserved in trees (Anderegg *et al*., 2016), among herbaceous angiosperms, hydraulic traits may be more evolutionarily labile.

### Conclusion

Our results highlight the critical role of phenology in shaping drought tolerance strategies among herbaceous species. Despite growing in the same environment, spring and late‐summer‐flowering species exhibited significant differences in leaf embolism resistance, SSM, and, to a lesser extent, TLP. Our data suggest that an annual lifecycle requires species to prioritize carbon gain at the risk of embolism‐induced mortality. While late‐summer‐flowering species had a wider SSM facilitating the avoidance of embolism, supporting a hydraulically safe strategy that supports long‐term persistence rather than immediate growth. Together, these patterns reveal a central ecological trade‐off where spring‐flowering species rely on speed and opportunism under more hydric early‐season conditions, whereas late‐summer species, especially perennials, emphasize hydraulic safety and long‐term persistence. These divergent strategies illustrate how drought response in herbaceous plants is tightly coupled with life history, flowering phenology, and ecology. Our findings contribute to a growing understanding of how herbaceous plants adapt to environmental stressors, emphasizing the role of leaf embolism resistance as a key driver of drought tolerance. Furthermore, as climate change continues to alter the timing and severity of droughts, understanding the link between phenology and hydraulic traits will be essential for predicting herbaceous species dynamics and resilience.

## Competing interests

None declared.

## Author contributions

IMR and SAMM contributed to the study's design. IMR collected data, conducted statistical analyses, and led the manuscript writing with help in all aspects from SAMM.

## Disclaimer

The New Phytologist Foundation remains neutral with regard to jurisdictional claims in maps and in any institutional affiliations.

## Supporting information


**Fig. S1** Distribution of species observations across the year.
**Fig. S2** Mean vulnerability curves for all spring flowering species.
**Fig. S3** Mean vulnerability curves for all later‐summer flowering species.
**Fig. S4** Mean P_12_, P_50_ and P_88_ across the canopy of *Solidago canadensis*.
**Table S1** Correlation table between anatomical and physiological traits across all species.
**Table S2** Correlation table between anatomical and physiological traits in spring flowering species.
**Table S3** Correlation table between anatomical and physiological traits in later‐summer flowering species.
Please note: Wiley is not responsible for the content or functionality of any Supporting Information supplied by the authors. Any queries (other than missing material) should be directed to the *New Phytologist* Central Office.

## Data Availability

Data are included in the manuscript and the Tables S1–S3 of the Supporting Information.
