# Supplementary material for: Temporally disjunct herbaceous species differ in leaf embolism resistance
Source: New Phytol. 2025 Jul 1;247(6):2630–46. doi: 10.1111/nph.70335 (PMC12371185; doi:10.1111/nph.70335)
Supplement: Supplementary file 1 — Fig. S1 Distribution of species observations across the year. Fig. S2 Mean vulnerability curves for all spring flowering species. Fig. S3 Mean vulnerability curves for all later‐summer flowering species. Fig. S4 Mean P12, P50 and P88 across the canopy of Solidago canadensis. Table S1 Correlation table between anatomical and physiological traits across all species. Table S2 Correlation table between anatomical and physiological traits in spring flowering species. Table S3 Correlation table between anatomical and physiological traits in later‐summer flowering species. Please note: Wiley is not responsible for the content or functionality of any Supporting Information supplied by the authors. Any queries (other than missing material) should be directed to the New Phytologist Central Office. [file NPH-247-2630-s001.docx]

**New Phytologist Supporting Information
Article title: Temporally disjunct herbaceous species differ in leaf embolism resistance
Authors: Ian M. Rimer and Scott A. M. McAdam
Article acceptance date: 10 June 2025**

**Supplementary Tables**

**Table S1:** Correlation table between leaf P_50_ (n=40) and measured anatomical trait (LMA), and OP, TLP, and the SSM (Pearson, R) across all 40 measured species. Stars denote significance, ‘***’ P<0.001, ‘**’ 0.001<P<0.01, ‘*’ 0.01<P<0.05.

|  | **P_50_** | **LMA** | **OP** | **TLP** |
| --- | --- | --- | --- | --- |
| **LMA** | *-0.22* |  |  |  |
| **OP** | *0.67 **** | *-0.14* |  |  |
| **TLP** | *0.67 **** | *-0.14* | *1.00 **** |  |
| **SSM** | *0.86 **** | *-0.20* | *0.20* | *0.20* |

**Table S2.** Correlation table between leaf P_50_ (n=20) and measured anatomical trait (LMA), and OP, TLP, and the SSM (Pearson, R) across all spring flowering species. Stars denote significance, ‘***’ P<0.001, ‘**’ 0.001<P<0.01, ‘*’ 0.01<P<0.05.

|  | **P_50_** | **LMA** | **OP** | **TLP** |
| --- | --- | --- | --- | --- |
| **LMA** | *-0.21* |  |  |  |
| **OP** | *0.83 **** | *-0.32* |  |  |
| **TLP** | *0.83 **** | *-0.32* | *1.00 **** |  |
| **SSM** | *0.83 **** | *-0.03* | *0.38* | *0.38* |

**Table S3.** Correlation table between leaf P_50_ (n=20) and measured anatomical trait (LMA), and OP, TLP, and the SSM (Pearson, R) across all late-summer flowering species. Stars denote significance, ‘***’ P<0.001, ‘**’ 0.001<P<0.01, ‘*’ 0.01<P<0.05.

|  | **P_50_** | **LMA** | **OP** | **TLP** |
| --- | --- | --- | --- | --- |
| **LMA** | *-0.21* |  |  |  |
| **OP** | *0.83 **** | *-0.32* |  |  |
| **TLP** | *0.83 **** | *-0.32* | *1.00 **** |  |
| **SSM** | *0.83 **** | *-0.03* | *0.38* | *0.38* |

**Supplementary Figures**

**
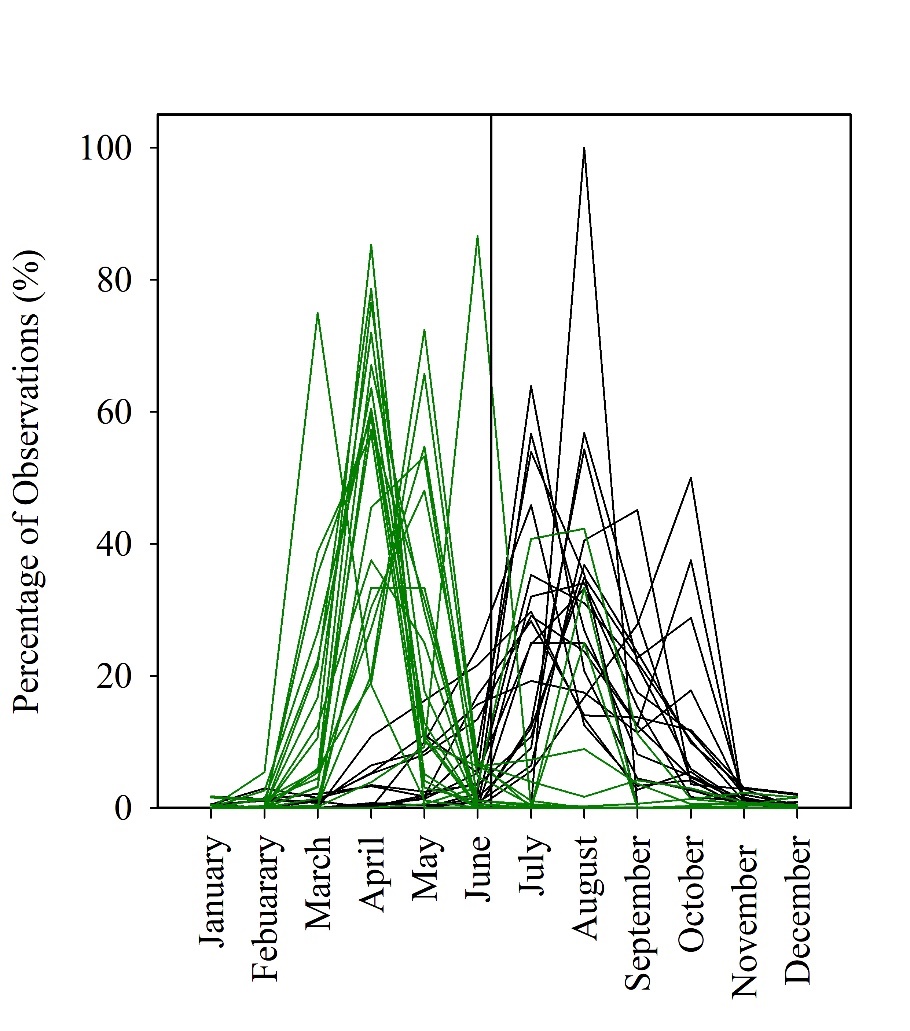
**

**Figure S1:** The normalized observations of species measured in this study were taken from publicly available datasets (https://www.inaturalist.org/) for the spring (green) and late-summer (black) flowering species. With observations of species being constrained to Central Indiana, set to only research-grade flower observations.


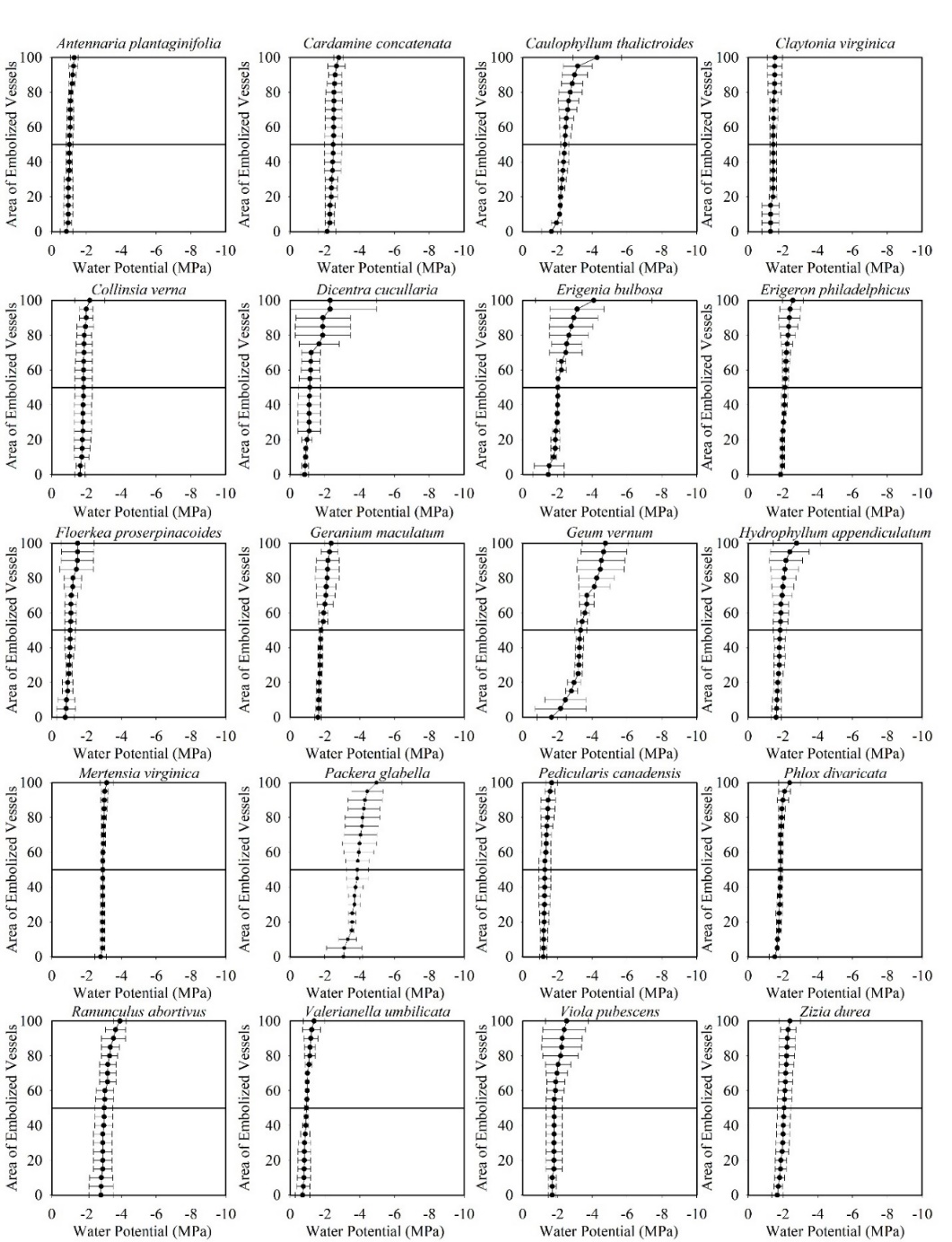


**Fig. S2:** Mean (±SD) vulnerability curves depicting the area of embolized xylem over decreasing Ψ_l_ (MPa) in the spring flowering herbaceous species. The horizontal line shows the point at which 50% of the xylem conduits have been embolized (P_50_).

**
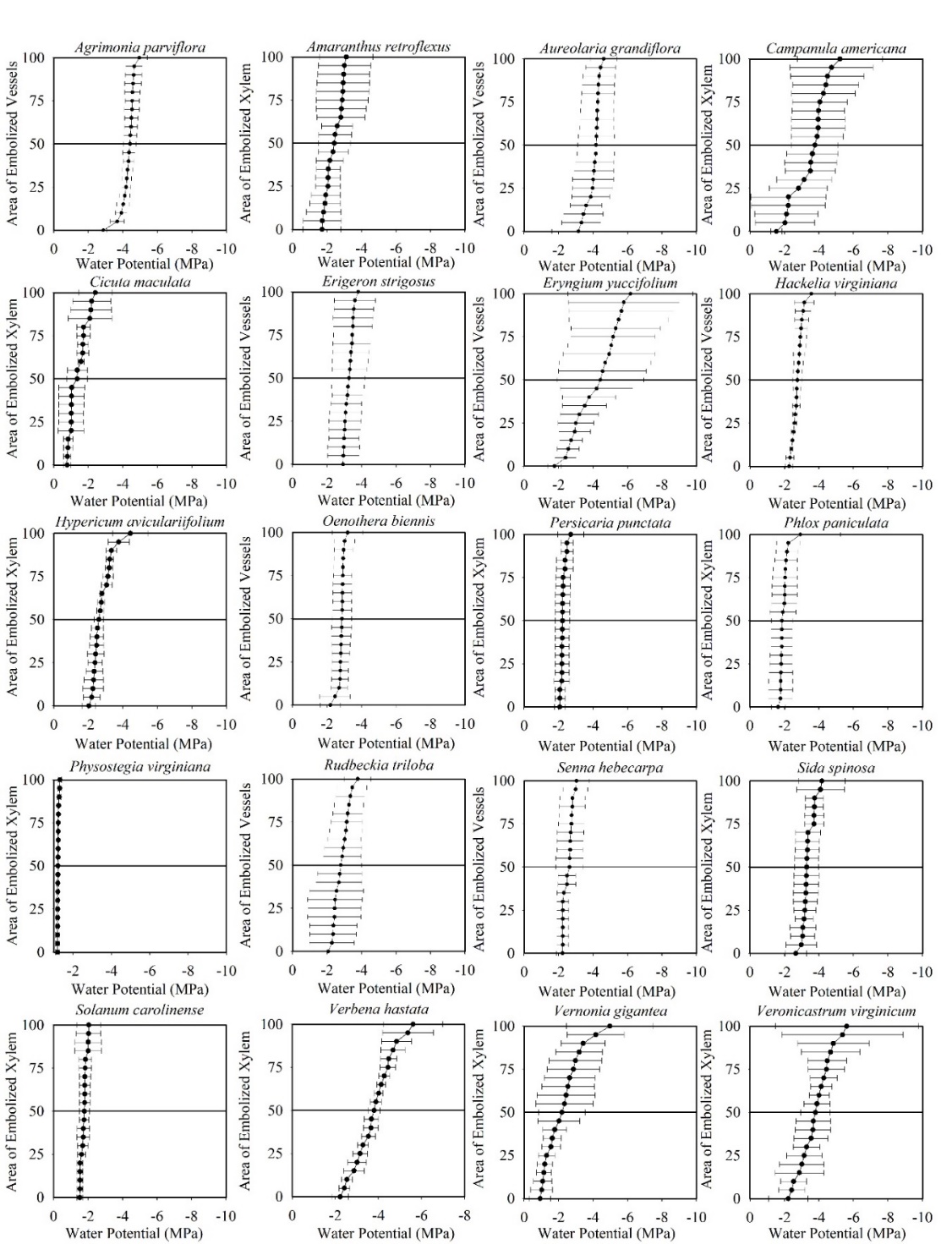
**

**Fig. S3:** Mean (±SD) vulnerability curves depicting the area of embolized xylem over decreasing Ψ_l_ (MPa) in late-summer flowering herbaceous species. The horizontal line shows the point at which 50% of the xylem conduits have been embolized (P_50_).


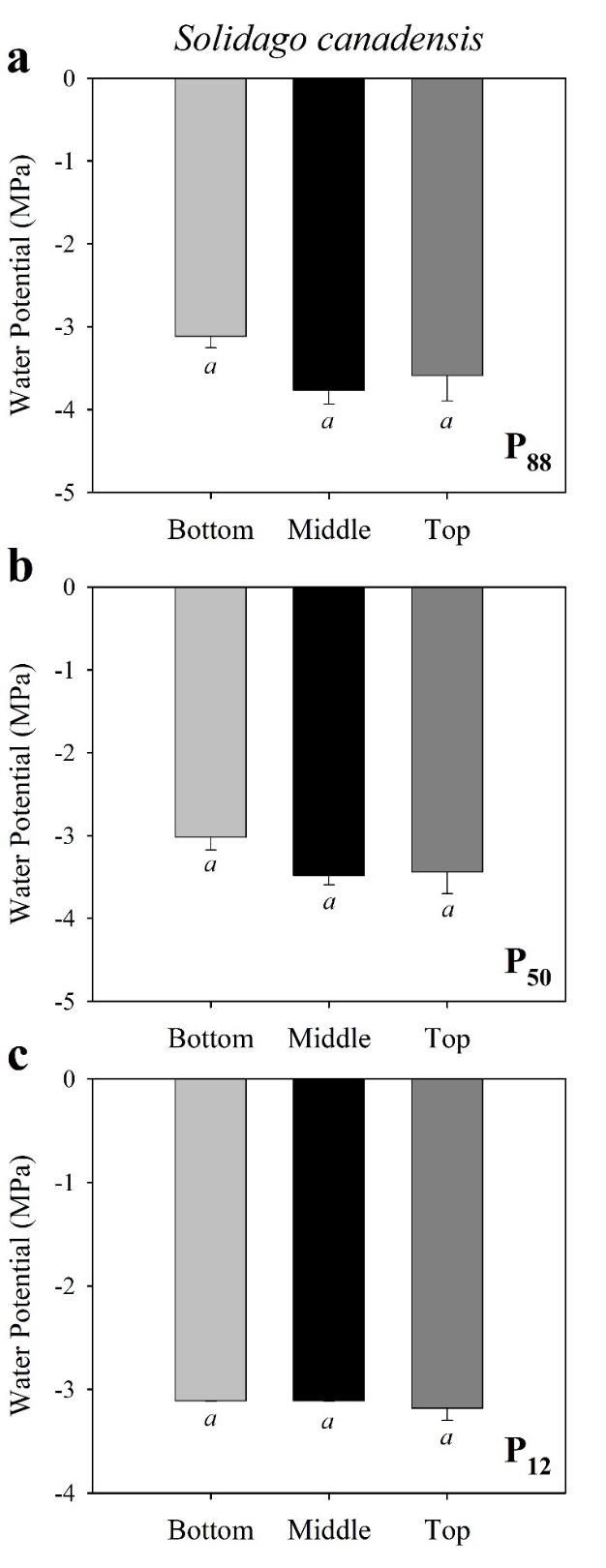


**Fig S4:** The mean (a) P_88_ (MPa, n=3 ± SE), (b) P_50_ (MPa, n=3 ± SE), and (c) P_12_ (MPa, n=3 ± SE) of a bottom, middle, and top leaf of *Solidago canadensis.* Significant differences between the bottom, middle, and top leaf for P_88_, P_50_, and P_12_ are denoted in lowercase italicized letters (ANOVA). Mean values can be found in Table 5.
